# Diterpenoid Tanshinones and Phenolic Acids from Cultured Hairy Roots of *Salvia miltiorrhiza* Bunge and Their Antimicrobial Activities

**DOI:** 10.3390/molecules16032259

**Published:** 2011-03-07

**Authors:** Jianglin Zhao, Jingfeng Lou, Yan Mou, Peiqin Li, Jianyong Wu, Ligang Zhou

**Affiliations:** 1 College of Agronomy and Biotechnology, China Agricultural University, Beijing 100193, China; 2 Department of Applied Biology and Chemical Technology, The Hong Kong Polytechnic University, Hung Hom, Kowloon, Hong Kong, China

**Keywords:** diterpenoid tanshinones, phenolic acids, cultured hairy roots, *Salvia miltiorrhiza* Bunge, antimicrobial activity

## Abstract

Four diterpenoid tanshinones and three phenolic acids were isolated from the crude ethanol extract of the cultured hairy roots of *Salvia miltiorrhiza* Bunge by bioassay-guided fractionation. By means of physicochemical and spectrometric analysis, they were identified as tanshinone ΙΙA (**1**), tanshinone Ι (**2**), cryptotanshinone (**3**), dihydrotanshinone Ι (**4**), rosmarinic acid (**5**), caffeic acid (**6**), and danshensu (**7**). These compounds were evaluated to show a broad antimicrobial spectrum of activity on test microorganisms including eight bacterial and one fungal species. Among the four tanshinones, cryptotanshinone (**3**) and dihydrotanshinone Ι (**4**) exhibited stronger antimicrobial activity than tanshinone ΙΙA (**1**) and tanshinone Ι (**2**). The results indicated that the major portion of the antimicrobial activity was due to the presence of tanshinones and phenolic acids in *S. miltiorrhiza* hairy roots, which could be used as the materials for producing antimicrobial agents for use in agricultural practice in the future.

## 1. Introduction

Plant tissue culture is based on the fact that plant cells are biosynthetically totipotent, with each cell retaining complete genetic information *in vitro*, and are thus capable of producing a range of metabolites found in the parent plant [1,2]. Compared with the traditional approach of whole plant cultivation in the natural environment or on agricultural farms, plant tissue culture in shake-flasks or bioreactors has the advantages of offering more well-controlled and sustainable culture systems without the limitations of natural factors such as geographical location and seasonal variation. It has been regarded as a promising way for mass production of valuable secondary metabolites, such as food additives, pharmaceuticals and nutraceuticals, antimicrobials and pesticides, particularly from those rare and slow-growing plant species [3,4,5,6].

**Figure 1:**
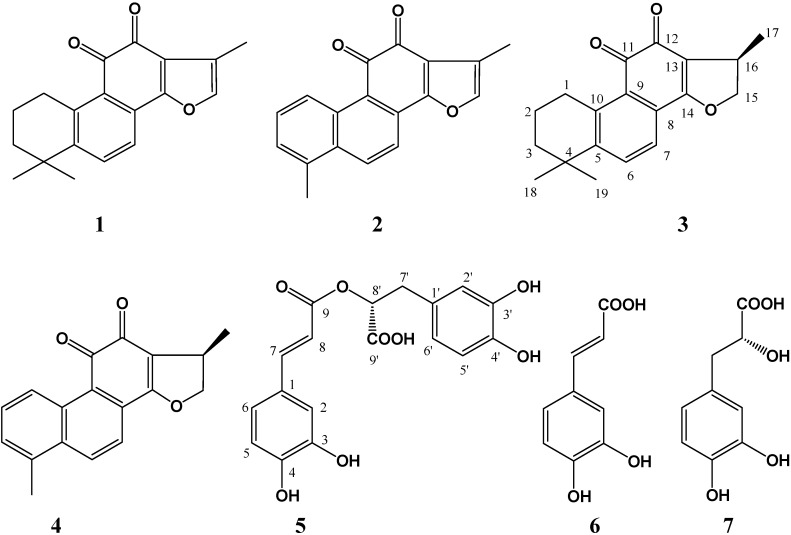
Chemical structures of the compounds: tanshinone IIA (**1**), tanshinone I (**2**), cryptotanshinone (**3**), dihydrotanshinone I (**4**), rosmarinic acid (**5**), caffeic acid (**6**), and danshensu (**7**).

*Salvia miltiorrhiza* Bunge (Lamiaceae) is an important and well-known medicinal plant. Its root, known as "Danshen" in Chinese, has been widely used in Traditional Chinese Medicine (TCM) for the treatment of menstrual disorders and cardiovascular diseases, as well as for the prevention of inflammation [7]. The main bioactive constituents of *S. miltiorrhiza* include water-soluble phenolic acids and lipophilic diterpenoid tanshinones. These bioactive compounds have been proven to have pronounced antioxidant, antibacterial, anticoagulant and antineoplastic activities, and show great potential applications in pharmaceutical and medicinal industry [8,9]. The hairy roots (or called transformed roots) of *S. miltiorrhiza* were transformed by the infection of plantlets with *Agrobacterium rhizogenes* containing Ri plasmid, and they have been established as a potential means for the production of tanshinone diterpenoids [10,11,12]. To the best of our knowledge, antimicrobial compounds in the cultured hairy roots of *S. miltiorrhiza* have not been investigated. The purpose of this study was to determine the antimicrobial components of *S. miltiorrhiza* hairy roots by bioassay-guided fractionation, as well as to evaluate antimicrobial activity of these compounds for their potential applications as antimicrobial agents.

## 2. Results and Discussion

### 2.1. Isolation and identification

Seven compounds were obtained from the crude ethanol extract of the hairy roots of *Salvia miltiorrhiza* by bioassay-guided fractionation. After comparing their physicochemical and spectral data with those reported in literature, they were identified as known compounds and confirmed as tanshinone ΙΙA (**1**) [13], tanshinone Ι (**2**) [13], cryptotanshinone (**3**) [13,14], dihydrotanshinone Ι (**4**) [13], rosmarinic acid (**5**) [15], caffeic acid (**6**) [16] and danshensu (**7**) [17] (Figure 1). All these compounds existing in the *Salvia miltiorrhiza* hairy root cultures were also confirmed by TLC and HPLC analysis using the corresponding standards as references. Of them, compounds **1**–**4** are tanshinone diterpenoids, and compounds **5**–**7** are phenolic acids. They have been previously isolated from the intact plant of *S. miltiorrhiza*, as well as other *Salvia* species [7,8,9]. 

### 2.2. Antimicrobial activity

The antimicrobial activities of these compounds were evaluated by micro-dilution-colorimetric and spore germination assays with the results listed in Table 1 and Table 2. Among the four tanshinones, both cryptotanshinone (**3**) and dihydrotanshinone I (**4**) exhibited strong antimicrobial activity. The minimum inhibitory concentration (MIC) values of cryptotanshinone (**3**) and dihydrotanshinone I (**4**) on all test bacteria ranged from 6.25 µg/mL to 100 µg/mL, and the median inhibitory concentration (IC_50_) values from 3.66 µg/mL to 57.38 µg/mL. MIC values of cryptotanshinone (**3**) and dihydrotanshinone I (**4**) on the spore germination of *M. oryzae* were 6.25 µg/mL and 3.13 µg/mL, respectively. Correspondingly, their IC_50_ values were 3.40 µg/mL and 0.91 µg/mL. Their antibacterial and antifungal activity was close to that of the positive controls (streptomycin sulfate or carbendazim). Both tanshinone IIA (**1**) and tanshinone I (**2**) were demonstrated to have moderate antimicrobial activity. According to the biosynthesis pathway of tanshinones in *S. miltiorrhiza* [18,19,20], cryptotanshinone (**3**) and dihydrotanshinone I (**4**) should be the precursors of tanshninone IIA (**1**) and tanshinone I (**2**), respectively. Cryptotanshinone (**3**) or dihydrotanshinone I (**4**) displayed stronger antimicrobial activity than that of tanshninone IIA (**1**) or tanshinone I **(2)** in this study. It can be speculated that the ethylenic linkage at C-15 and C-16 positions along with the methyl group conformation at the C-16 position were important for the antimicrobial activity of the tanshinones. Our results were in accord with previous reports [21,22]. Many tanshinones have been reported to exhibit antimicrobial and cytotoxic activities [9,20,23].

By comparison with the isolated tanshinones **1**–**4**, the three phenolic acids **5**–**7** exhibited weak antimicrobial activity on the test microorganisms. Antimicrobial activity of rosmarinic acid (**5**) was similar to that of caffeic acid (**6**). Danshensu (**7**) exhibited the weakest activity among three isolated phenolic acids. It is possible that the ethylenic linkage at C-7 and C-8 positions is critical for the antimicrobial activity of these phenolic acids. Both rosmarinic acid (**5**) and danshensu (**7**) should be considered derivatives of caffeic acid (**6**) based on the biosynthesis pathway [24]. Phenolic acids with a variety of bioactivities, including antimicrobial, antioxidant, anti-thrombosis, anti-hypertension, antivirus and antitumor properties, are widely distributed in the plant kingdom [24,25].

**Table 1:** MIC values of the compounds **1**–**7** obtained from the cultured hairy roots of *S. miltiorrhiza* on test microorganisms.

Micro-organism	MIC (μg/mL)							
	1	2	3	4	5	6	7	CK^+^
*A. t*	50	25	12.5	6.25	100	100	150	6.25
*E. c*	50	50	12.5	25	100	100	150	25
*P. l*	25	25	12.5	12.5	50	50	100	12.5
*R. s*	50	50	12.5	12.5	50	100	100	12.5
*X. v*	25	25	6.25	12.5	50	100	100	12.5
*B. s*	50	50	25	25	100	150	150	50
*S. a*	200	150	100	100	200	200	>200	100
*S. h*	100	100	50	50	150	150	200	50
*M. o*	50	12.5	6.25	3.13	150	200	>200	6.25

Note: *A. t.*, *A. tumefaciens*. *E. c.*, *E. coli*. *P. l.*, *P. lachrymans*, *R. s.*, *R. solanacearum*. *X. v.*, *X. vesicatoria*. *B. s.*, *B. subtilis*. *S. a.*, *S. aureus*. *S. h.*, *S. haemolyticus*. *M. o.*, *M. oryzae*. MIC, minimum inhibitory concentration. The positive controls (CK^+^) on test bacteria and fungus were streptomycin sulfate and carbendazim, respectively.

**Table 2:** IC_50_ values of the compounds **1**–**7** obtained from the cultured hairy roots of *S. miltiorrhiza* on test microorganisms.

Micro-organism	IC_50_ (μg/mL)							
	1	2	3	4	5	6	7	CK^+^
*A. t*	31.25 ± 0.56^d^	13.56 ± 0.26^e^	7.28 ± 0.35^f^	4.03 ± 0.09^g^	62.78 ± 1.21^c^	65.32 ± 0.76^b^	87.36 ± 1.02^a^	3.83 ± 0.05^g^
*E. c*	27.54 ± 0.38^d^	20.29 ± 0.43^e^	7.83 ± 0.51^g^	11.17 ± 0.13^f^	52.15 ± 0.82^c^	57.28 ± 0.63^b^	96.75 ± 1.36^a^	7.55 ± 0.18^g^
*P. l*	16.32 ± 0.25^d^	11.18 ± 0.37^e^	7.32 ± 0.62^g^	9.10 ± 0.23^f^	27.03 ± 0.51^c^	32.65 ± 0.35^b^	64.02 ± 0.73^a^	6.82 ± 0.10^g^
*R.s*	35.36 ± 0.52^c^	31.58 ± 0.85^e^	7.12 ± 0.23^f^	7.78 ± 0.26^f^	33.15 ± 0.63^d^	63.27 ± 0.79^b^	68.25 ± 0.64^a^	6.75 ± 0.06^f^
*X. v*	14.43 ± 0.31^d^	11.09 ± 0.29^e^	3.66 ± 0.16^h^	7.57 ± 0.51^f^	38.56 ± 0.49^c^	57.23 ± 0.61^b^	72.38 ± 0.89^a^	5.72 ± 0.12^g^
*B. s*	32.59 ± 0.67^d^	27.54 ± 0.45^e^	15.48 ± 0.34^g^	17.61 ± 0.38^f^	73.86 ± 0.92^c^	91.85 ± 1.04^b^	94.32 ± 1.16^a^	27.35 ± 1.06^e^
*S. a*	108.87 ± 1.83^c^	83.06 ± 1.32^d^	50.25 ± 1.42^g^	57.38 ± 0.92^f^	131.82 ± 1.58^b^	142.36 ± 2.13^a^	nd	65.98 ± 1.32^e^
*S. h*	73.34 ± 1.21^d^	61.81 ± 1.05^e^	28.87 ± 0.88^h^	35.56 ± 1.23^f^	83.61 ± 1.32^c^	102.58 ± 1.83^b^	164.26 ± 2.35^a^	31.94 ± 1.18^g^
*M. o*	33.02 ± 0.87^c^	6.10 ± 0.64^d^	3.40 ± 0.12^e^	0.91 ± 0.07^f^	92.53 ± 1.36^b^	128.35 ± 1.25^a^	nd	2.86 ± 0.16^e^

Note: The abbreviations for the microorganism names were the same as those in Table 1. IC_50_, median inhibitory concentration. The positive controls (CK^+^) on test bacteria and fungus were streptomycin sulfate and carbendazim, respectively. The 'nd' means not detected. Mean ± standard deviation of three independent experiments (three replicates for each treatment). Different letters (*i.e.*, a–h) indicated significant differences among the test compounds on a certain microorganism at p = 0.05 level.

## 3. Experimental

### 3.1. General

Melting points of the compounds were measured on an XT4-100B microscopic melting-point apparatus (Tianjin Tianguang Optical Instruments Company, China) and uncorrected. NMR spectra were recorded on a Bruker Avance DRX-500 (^1^H at 500 MHz and ^13^C at 125 MHz) spectrometer using tetramethylsilane (TMS) as an internal standard, and chemical shifts were recorded in ppm as *δ* values. ESI-MS spectra were recorded on a Bruker Esquire 6000 LC/MS spectrometer. Both silica gel (200–300 mesh) for column chromatography (CC) and silica gel GF_254_ (10–20 mm) for thin layer chromatography (TLC) were the products of Qingdao Marine Chemical Company, China. The Sephadex LH-20 and silica gel RP-18 were purchased from Pharmacia Biotech, Sweden. The microplate spectrophotometer (PowerWave HT, BioTek Instruments, USA) was employed to measure the light absorption value. Streptomycin sulfate and carbendazim were purchased from Sigma-Aldrich (USA). 3-(4,5-Dimethylthiazol-2-yl)-2,5-dephenyl tetrazolium bromide (MTT) was purchased from Amresco (USA). All other chemicals and reagents were of analytical grade.

### 3.2. Plant material

The hairy roots of *S. miltiorrhiza* Bunge were obtained after the infection of the plantlets with *Agrobacterium rhizogenes* ATCC 15834 containing an Ri plasmid [11]. The liquid culture of *S. miltiorrhiza* hairy roots was carried out in 250-mL Erlenmeyer flasks on an orbital shaker at 110–120 rpm and 25 °C in the dark, and each flask was filled with 50 mL of liquid hormone-free MS medium [26] including 8 g/L of agar, 30 g/L of sucrose and 0.5 g/L of casein hydrolysate but without ammonium nitrate. The culture period was 35 days, and then the *S. miltiorrhiza* hairy roots were harvested and left to dry in the shade at room temperature to a constant weight.

### 3.3. Extraction, fractionation and identification

The air-dried and powdered *S. miltiorrhiza* hairy roots (450 g) were ready for extraction and fractionation of the antimicrobial compounds. First, they were extracted three times with 95% ethanol (1.5 L) at room temperature under sonication for 60 min. After removal of the solid, the combined filtrate was evaporated under vacuum at 50 °C to dryness, and a total of 24.5 g brown residue was obtained. The crude ethanol extract was firstly subjected to a cut-column chromatography (CC) over silica gel (200–300 mesh) eluted with CHCl_3_-MeOH (10:0.5, v/v) to obtain six fractions (A, B, C, D, E and F) according to thin layer chromatography test. Fraction A (0.48 g) was re-subjected to silica gel column chromatography eluted with cyclohexane-acetone (from 1:0 to 0:1, v/v) to give 80 sub-fractions. Fraction A-12 to A-16 were combined and purified by recrystallization to afford **1** (21.0 mg). Fraction A-28 to A-32 were combined, and further purified over Sephadex LH-20 and reverse phase chromatography (RP-18) to afford **2** (11.2 mg). Fraction A-40 to A-45 were combined, and further purified over Sephadex LH-20 and recrystallization to afford **3** (15.6 mg). Fraction C (0.15 g) was purified over Sephadex LH-20 and recrystallization to yield **4** (18.5 mg). Fraction D (0.28 g) was re-subjected to silica gel column eluted with CHCl_3_-MeOH-HCOOH (from 1:0:0 to 0:1:0.01, v/v) to give 60 subfractions. Fraction D-30 to D-35 were combined, and further purified over Sephadex LH-20 and recrystallization to afford **5** (25.8 mg). Fraction E (0.65 g) was re-subjected to silica gel column eluted with CHCl_3_-MeOH-HCOOH (from 1:0:0 to 0:1:0.01, v/v) to give 60 subfractions. Fraction E-25 to E-28 was combined and further purified over Sephadex LH-20 and recrystallization to yield **6** (35.6 mg). Fraction E-42 to E-46 were combined, and further purified over Sephadex LH-20 and reverse phase chromatography (RP-18) to afford **7** (18.2 mg). Their physicochemical and spectrometric data were determined and compared with those reported in the literature.

### 3.4. Antibacterial activity assay

Five Gram-negative (*Agrobacterium tumefaciens* ATCC 11158, *Escherichia coli* ATCC 29425, *Pseudomonas lachrymans* ATCC 11921, *Ralstonia solanacearum* ATCC 11696 and *Xanthomonas vesicatoria* ATCC 11633) and three Gram-positive (*Bacillus subtilis* ATCC 11562, *Staphylococcus aureus* ATCC 6538 and *Staphylococcus haemolyticus* ATCC 29970) bacteria were selected for antibacterial activity assay. They were grown in liquid Luria-Bertani (LB) medium (yeast extract 5 g/L, peptone 10 g/L, NaCl 5 g/L, pH 7.0) overnight at 28 °C, and the diluted bacterial suspension (1 × 10^6^ cfu/mL) was ready for detection. A modified micro-dilution-colorimetric assay by using the chromogenic reagent 3-(4,5-dimethylthiazol-2-yl)-2,5-dephenyl tetrazolium bromide (MTT) was used to detect the antibacterial activity of these compounds according to our previous report [27]. Briefly, the test compound was dissolved in acetone at an initial concentration of 8.0 mg/mL. Then it was diluted with 30% acetone to obtain concentrations ranging from 7.8 μg/mL to 2.0 mg/mL. Test sample solutions (10 µL) and prepared bacterial suspension (90 µL) containing 1 × 10^6^ cfu/mL were added into each well of the 96-well microplate. Each well of the negative control contained 90 µL of the inoculum (1 × 10^6^ cfu/mL) and 10 µL of 30% acetone. Streptomycin sulfate was used as the positive control. After the plates were agitated to mix the contents of the wells using a plate shaker and incubated in the dark at 28 °C for 24 h, 10 µL of MTT (5 mg/mL in 0.2 mol/L, pH 7.2, phosphate-buffered saline, PBS) was added into each well, and the plates were incubated for another 4 h. The minimum inhibitory concentration (MIC) value was defined as the lowest sample concentration that inhibited visible growth of the test bacterium, as indicated by the MTT staining. Only living microorganisms can convert MTT to formazan, and a blue color appeared in the well [28].

To further determine the median inhibitory concentration (IC_50_) value of each sample, the above MTT stained suspension was centrifuged at 1,500 g for 20 min. Then the supernatant was aspirated, 150 µL of dimethyl sulfoxide (DMSO) was added into each well, and the colored formazan products were extracted for 30 min. After complete extraction, the plate was centrifuged at 1,500 g for another 20 min, and then 100 µL of the supernatant in each well was transferred to a corresponding well of another 96-well microplate to measure their light absorption values at wavelength 510 nm using a microplate spectrophotometer. The percentage (%) of the bacterial growth inhibition was determined as [(*A*_c_-*A*_t_)/*A*_c_] × 100, where *A*_c_ was an average of three replicates of light absorption values at wavelength 510 nm of the negative controls, and *A*_t_ was the average of three replicates of light absorption values of the samples. The IC_50_ value was calculated using the linear relation between the inhibitory probability and concentration logarithm according to the method of Sakuma [29].

### 3.5. Antifungal activity assay

Rice blast fungus, *Magnaporthe oryzae* (P131), was kindly provided by Prof. Youliang Peng from the Department of Plant Pathology, China Agricultural University. It was maintained on oatmeal-tomato agar (oatmeal 30 g/L, tomato juice 150 mL/L, and agar 20 g/L) at 25 °C. A spore germination assay was employed to detect the antifungal activity of these compounds. Briefly, the spores were prepared from 7-day-old cultures of *M. oryzae*, according to our previous reports [30,31]. The test compound-acetone solution (25 µL) was mixed with an equivalent volume of fungal spore suspension containing 2 × 10^6^ spores/mL. The mixture was then placed on separate concave glass slides. The final compound concentrations ranged from 0.78 µg/mL to 200 µg/mL in 5% (v/v) acetone. The negative control was 5% acetone, and the positive control was carbendazim with concentrations ranging from 0.78 µg/mL to 50 µg/mL. Three replicates were used for each treatment. Slides containing the spores were incubated in a moist chamber at 25 °C for 7 h. Each slide was then observed under the microscope for spore germination status. About 100 spores per replicate were observed to detect spore germination. The percentage (%) of spore germination inhibition was determined as [(*G*_c_-*G*_t_)/*G*_c_] × 100, where *G*_c_ is an average of three replicates of germinated spore number in the negative control, and *G*_t_ is an average of three replicates of germinated numbers in the treated sets. The IC_50_ value calculation for the spore germination inhibition was the same as that for antibacterial activity assay. The MIC value on the spore germination was defined as the lowest sample concentration that inhibited visible spore germination. If the length of germ tube was longer than that of spore diameter, the spore was considered to be germinated.

## 4. Conclusions

In this study, we have reported the isolation and identification of four diterpenoid tanshinones and three phenolic acids from the cultured hairy roots of *S. miltiorrhiza*. It was proved that the cultured hairy roots maintained their biosynthetic totipotency. The obtained tanshinones and phenolic acids exhibited a broad antimicrobial spectrum of activity, especially on plant pathogens. They could be the main antimicrobial compounds in the hairy roots of *S. miltiorrhiza*. The results provide additional data for the use of *S. miltiorrhiza* hairy roots as sources for producing antimicrobial agents, especially those applicable in agricultural practice in the future. By means of biotechnology, the production of bioactive compounds from hairy roots of *S. miltiorrhiza* instead of naturally growing roots could be an alternative and promising way. It also implies that both tanshinones and phenolic acids may play important roles in the defense system of *S. miltiorrhiza* which need to be clarified. Other issues including the action mechanisms of these compounds on microorganisms, and efficient strategies for increasing their content and yield in the hairy roots, as well as their preparation in large scale also need to be further studied.
